# Quantum chemical calculations of nitrosamine activation and deactivation pathways for carcinogenicity risk assessment

**DOI:** 10.3389/fphar.2024.1415266

**Published:** 2024-07-17

**Authors:** Andreas H. Göller, Sandra Johanssen, Adam Zalewski, Verena Ziegler

**Affiliations:** ^1^ Computational Molecular Design, Bayer AG, Pharmaceuticals, Wuppertal, Germany; ^2^ Industrial Chemicals and Marketed Products, Bayer AG, Pharmaceuticals, Berlin, Germany; ^3^ Genetic and Computational Toxicology, Bayer AG, Pharmaceuticals, Berlin, Germany

**Keywords:** nitrosamines, quantum chemical calculations, mutagenicity, carcinogenicity, activation pathway

## Abstract

N-nitrosamines and nitrosamine drug substance related impurities (NDSRIs) became a critical topic for the development and safety of small molecule medicines following the withdrawal of various pharmaceutical products from the market. To assess the mutagenic and carcinogenic potential of different N-nitrosamines lacking robust carcinogenicity data, several approaches are in use including the published carcinogenic potency categorization approach (CPCA), the Enhanced Ames Test (EAT), *in vivo* mutagenicity studies as well as read-across to analogue molecules with robust carcinogenicity data. We employ quantum chemical calculations as a pivotal tool providing insights into the likelihood of reactive ion formation and subsequent DNA alkylation for a selection of molecules including e.g., carcinogenic N-nitrosopiperazine (**NPZ**), N-nitrosopiperidine (**NPIP**), together with N-nitrosodimethylamine (**NDMA**) as well as non-carcinogenic N-nitrosomethyl-tert-butylamine (**NTBA**) and bis (butan-2-yl) (nitros)amine (**BBNA**). In addition, a series of nitroso-methylaminopyridines is compared side-by-side. We draw comparisons between calculated reaction profiles for structures representing motifs common to NDSRIs and those of confirmed carcinogenic and non-carcinogenic molecules with *in vivo* data from cancer bioassays. Furthermore, our approach enables insights into reactivity and relative stability of intermediate species that can be formed upon activation of several nitrosamines. Most notably, we reveal consistent differences between the free energy profiles of carcinogenic and non-carcinogenic molecules. For the former, the intermediate diazonium ions mostly react, kinetically controlled, to the more stable DNA adducts and less to the water adducts via transition-states of similar heights. Non-carcinogenic molecules yield stable carbocations as intermediates that, thermodynamically controlled, more likely form the statistically preferred water adducts. In conclusion, our data confirm that quantum chemical calculations can contribute to a weight of evidence approach for the risk assessment of nitrosamines.

## Introduction

N-nitrosamines have been discovered as impurities in medicinal products containing active pharmaceutical ingredients (API) such as valsartan, ranitidine, varenicline and metformin (Snodin and Elder, 2019; Glowienke et al., 2022). Many N-nitrosamines have been shown or are assumed to be potent mutagens and/or carcinogens (IARC, 1980). In contrast to other mutagenic and carcinogenic impurities that are in the scope of the ICH M7 (R2) guideline (ICH M7 and R2, 2023), N-nitrosamines belong to the cohort of concern, i.e., structural classes of highly potent carcinogens for which acceptable intakes (AI) would likely be lower than the threshold of toxicological concern (TTC) of 1,500 ng/day.

The detection of N-nitrosodimethylamine (**NDMA**), N-nitrosodiethylamine (**NDEA**) and N-nitroso-N-methyl-4-aminobutyric acid (**NMBA**) in several medicinal products at levels higher than their AIs led to recalls from the markets (EMA, 2018; FDA, 2018) and health authorities (HA) have issued specific guidance (FDA, 2021; EMA, 2024) for marketing authorization holders to request a detailed analysis of all chemically manufactured drug substances (DS) and drug products (DP) for the potential presence of N-nitrosamines. This revealed that not only small dialkyl N-nitrosamines but also nitrosamine drug substance related impurities (NDSRIs) are formed in medicinal products.

N-nitrosamines mainly originate from nitrosation of secondary amine groups of commonly used excipients or remaining traces of chemical synthesis reagents as well as APIs (Ponting and Foster, 2023). High effort has been made to mitigate N-nitrosamine levels in medicinal products (fda.gov, 2022) however, analytical testing often remains challenging as extremely low levels need to be detected and quantified (Dobo et al., 2022).

Nitrosamines are generally activated by cytochrome P450 (CYP) enzymes, i.e., mainly by CYP2E1 (Bellec et al., 1996) but also by other isoforms (Yamazaki et al., 1992; Chowdhury et al., 2012) within the human body initiating a series of spontaneous transformations concluding in reactive diazonium ions (described in detail below), causing DNA alkylation and eventually resulting in tumor formation. In particular, recent research revealed that molecules such as NDMA are prone to cause base substitution mutations such as GC→AT common to many cancer types (Schrenk et al., 2023). The specific metabolic conditions needed for nitrosamine detection have also led to some of them displaying negative results in the standard Ames test despite being rodent cancer bioassay positive (Thomas et al., 2024).

Acceptable intakes for specific N-nitrosamines are usually derived from TD50 values determined in animal carcinogenicity studies. For some N-nitrosamines, such as **NDMA** and **NBDA** this data is available (carcdb, 2022). Even though N-nitrosamines are often considered to be highly potent carcinogens, the TD50s span a broad range with regard to potency (Thresher et al., 2020).

The potency of small N-nitrosamine impurities APIs led to a widespread concern that NDSRIs could be also potent carcinogens. At the same time, carcinogenicity data for NDSRIs is sparse rendering the derivation of acceptable intakes difficult. This necessitates the development of alternative data sources informing marketing authorization holders and authorities alike about the safety of these impurities.

The current approaches to infer the safe levels of N-nitrosamines in absence of reliable *in vivo* data are based on thoroughly investigated structure-activity relationships linking various accompanying structural features to decreases or increases in their carcinogenic potency (Cross and Ponting, 2021). In addition, read-across for relevant substructures in an NDSRI for which carcinogenicity data are available is a conservative way to assess the carcinogenic potency (Cross and Ponting, 2021; EMA, 2022).

To complement the many ongoing initiatives of obtaining data on mutagenic and carcinogenic potential of nitrosamines, a thorough and detailed quantum chemical protocol has been established by Wenzel and colleagues (Wenzel et al., 2022). After slight adaptations, we use this to calculate activation profiles of nine molecules selected based on the availability of reference *in vivo* carcinogenicity data and presence of structural motifs commonly found in drug-like substances. We chose to abstain from using Ames mutagenicity data as reference due to the aforementioned insensitivity of the standard protocols to some N-nitrosamines as well as the fact that Ames testing does not quantify the mutagenic potency of compounds making it difficult to distinguish strong and weak mutagens. We reveal and rationalize consistent patterns helping to discriminate the reaction profiles of carcinogenic and non-carcinogenic substances. It is our hope that this method complements the growing toolbox of reliable, efficient methods enabling unambiguous assessment of N-nitrosamine substances considering animal welfare and time of *in vivo* experiments.

## Materials and methods

Quantum chemical calculations provide reliable physics-based first-principles information on reaction profiles and by this allow for the description of the stepwise activation or respectively deactivation of N-nitrosamines (assuming metabolic activation took place) via their reaction intermediates and transition states, thus quantifying the thermodynamic and kinetic parameters of the reaction pathways.

### Computational methods

The computational protocols used here follow the work initially published by Wenzel et al. (2022) but were slightly adapted to adhere to commonly accepted best practices (Goerigk and Grimme, 2011; Bursch et al., 2022). All semiempirical calculations were performed using the software XTB version 6.4.1 (github, 2022), conformational searches were done with CREST, (Grimme, 2019), and density functional theory calculations were done with ORCA, (Neese, 2012) version 5.0.3. Calculations were performed applying the workflow software WEASEL (WEASEL, 2023) by Faccts (FACCTS, 2022) on 32 core 3.9 GHz Intel CPU nodes with Redhat Linux 7.2 or 8.0 operating system.

Conformational searches were performed for all ground-state structures with CREST for conformer generation, followed by pre-optimization with GFN2-xTB (Bannwarth et al., 2019) with ALPB (Ehlert et al., 2021) solvent model and an energy filter of 6.0 kcal/mol, followed by geometry optimization with BP86 (Becke, 1988) and def2-TZVP(-F) basis set (Weigend and Ahlrichs, 2005) with D3 dispersion correction (Grimme et al., 2010) and CPCM solvent model (Barone and Cossi, 1998) with an energy filter of 5.0 kcal/mol, and finally energy calculations with ωB97X-V/def2-TZVP (Mardirossian and Head-Gordon, 2014) (energy filter of 4.0 kcal/mol).

The lowest energy conformer was then optimized according to Wenzel et al. with the B3LYP (Becke, 1993) functional and 6-311+G (d,p) basis set (Ditchfield et al., 1971) (enlarged to triple zeta basis for consistency reasons). Contrary to the previous work, D3 dispersion corrections were applied following the best-practices described by Grimme et al. (Bursch et al., 2022), and calculations were done using the SMD (Marenich et al., 2009) continuum solvent model. Vibrational frequency analysis was done on the same level of theory, and the correct numbers of imaginary frequencies and vibrational modes (0 for ground states and 1 for transition states) were visually inspected in Schrödinger Maestro (Schrodinger LLC, 2023). Finally, energy calculations were done with the M06-2x-D3 functional (Zhao and Truhlar, 2008) and the aug-cc-pVTZ basis set (Krishnan et al., 1980) with SMD solvent model. As mentioned by Wenzel et al., the M06-2x functional is known to be one the most accurate DFT methods to describe main group thermochemistry and kinetic parameters. (Marenich et al., 2009; Bhattacharjee and Shukla, 2014).

In seldom cases where the topology of the structures changed either in the conformational search or during the geometry optimization with the triple zeta diffuse basis set, semiempirical GFN2-xTB pre-optimizations were done applying the constraint -keeptopology in the respective WEASEL workflow.

Initial geometries for the transition state calculations were obtained either by educated guess or by automated fine-grained (0.05 Å) relaxed surface scans of the expected reaction pathways (both roads were always explored), as implemented in ORCA. The respective transition state structures were verified by visual inspection of the vibrational modes for the imaginary frequency obtained and further confirmed by intrinsic reaction coordinate (IRC) calculations.

Solution-phase Gibbs free energies were obtained as the sum of the final single-point energy, SMD solvation and the thermal corrections at 298 K from the vibrational analysis, following the established procedures, as presented extensively in the publication by Wenzel et al. (2022).

Chemical 3D structure depictions were created with Schrodinger Maestro (Schrodinger LLC, 2023). The activation profile diagrams were created in Microsoft Excel using a template provided by John Keller (Template received, 2022).

### Molecules

**SCHEME 1:**
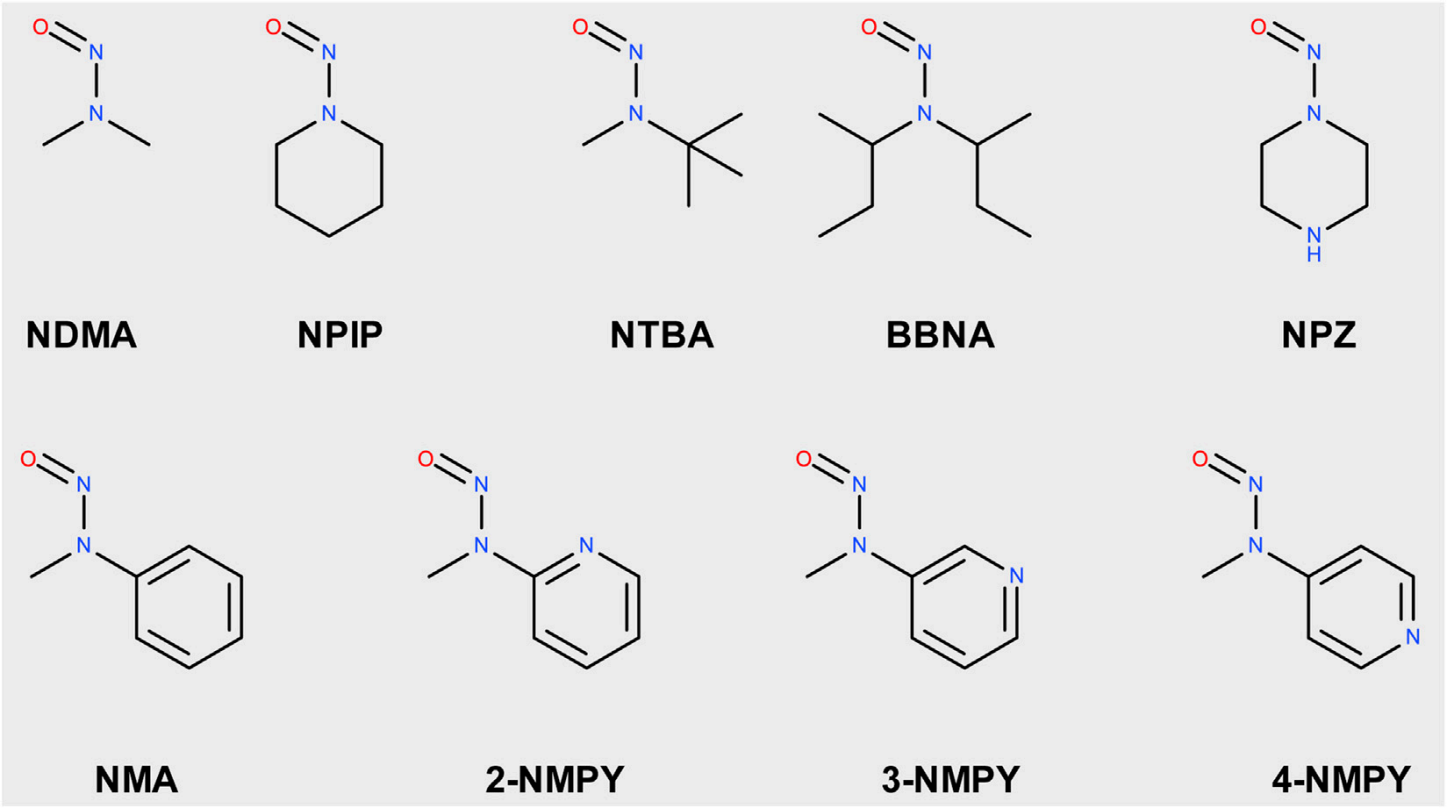
Structures of N-nitrosamines; NDMA: N-nitrosodimethylamine; NPIP: N-nitrosopiperidine; NTBA: N-nitrosomethyl-tert-butylamine; BBNA: bis(butan-2-yl) (nitros)amine; N-Nitrosopiperidine; NPZ: N-nitrosopiperazine; NMA: N-nitroso-N-methylaniline; 2-NMPY: N-nitroso-N-methyl-2-aminopyridine; 3-NMPY: N-nitroso-N-methyl-3-aminopyridine; 4-NMPY: N-nitroso-N-methyl-4-aminopyridine.

The nine molecules discussed in this publication are depicted in Scheme 1. The first set consists of the small aliphatic N-nitrosodimethylamine **NDMA** and N-nitrosopiperidine **NPIP** which are cohort of concern compounds with low Acceptable Intake (AI) values (below 1,500 ng/d). Both compounds are described by Wenzel et al. (2022) as AC01 and CYC06. They are here used (i) as reference of reaction profiles of low AI compounds and (ii) to assess if the adjustments to the computational protocol change the reaction profiles. N-nitrosomethyl-tert-butylamine **NTBA**, bis(butan-2-yl) (nitros)amine **BBNA**, and N-nitrosopiperazine **NPZ** are derivatives with substituents leading to no or low carcinogenicity. Finally, compounds N-nitroso-N-methylaniline **NMA** (CS01 in Wenzel et al.), 2-nitroso-methylaminopyridine **2-NMPY**, N-nitroso-N-methyl-3-aminopyridine **3-NMPY** and 4-nitroso-methylaminopyridine **4-NMPY** comprise a series with aromatic moieties that only differ by the existence and position of one nitrogen atom which determines experimentally observed carcinogenicity (carcdb, 2022). Taken together, this set of molecules allowed confirming that the adapted protocol complied with the results from Wenzel et al. while also adding NDSRI-representative motifs (particularly non-carcinogenic ones) that have yet not been investigated to this degree. Table 1 provides qualitative (positive/negative) and quantitative experimental carcinogenicity data for the selected substances. It also exemplifies discrepancies between various sources of acceptable intakes (e.g., 70-fold difference between limits suggested for **NPZ**) stemming from the complexity of *in vivo* data, underscoring the need of unambiguous and reproducible quantitative methods to assess nitrosamines.

**TABLE 1:** Carcinogenicity and acceptable intakes (AI) for the nine investigated N-nitrosamines; NDMA: N-nitrosodimethylamine; NPIP: N-nitrosopiperidine; NTBA: N-Nitrosomethyl-tert-butylamine; BBNA: bis(butan-2-yl) (nitros)amine; N-Nitrosopiperidine; NPZ: N-nitrosopiperazine; NMA: N-nitroso-N-methylaniline; 2-NMPY: N-nitroso-N-methyl-2-aminopyridine; 3-NMPY: N-nitroso-N-methyl-3-aminopyridine; 4-NMPY: N-nitroso-N-methyl-4-aminopyridine; CoC: Cohort of Concern.

#	name	CAS no	Carcino-genicity (carcdb, 2022)	Health authorities AI (ng/day)	Citation	AI (ng/day) Bercu et al. (2023)
1	NDMA	62-75-9	positive (CoC)	96	EMA, FDA, HC	145
2	NPIP	100-75-4	positive (CoC)	1,300	EMA, HC	1,300
3	NTBA	2504-18-9	negative	-	-	-
4	BBNA	5350-17-4	negative	-	-	-
5	NPZ	5632-47-3	positive	400	EMA, HC	28,500
6	NMA	614-00-6	positive	-	(Boyland et al., 1964; Kroeger-Koepke et al., 1983; Schmahl, 1975)	-
7	2-NMPY	16,219-98-0	positive	-	Preussmann et al. (1979)	-
8	3-NMPY	69,658-91-9	negative	-	Preussmann et al. (1979)	-
9	4-NMPY	16,219-99-1	negative	-	Preussmann et al. (1979)	-

### Activation pathway

**FIGURE 1:**
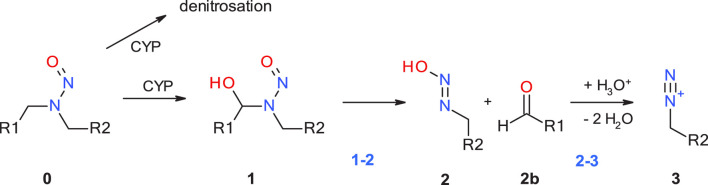
Linear part of the activation pathway of N-nitrosamines in case of aliphatic nitrosamines. CYP: Cytochrome P450.

The first step of the activation pathway as mapped out in Figure 1 is hydroxylation of an aliphatic carbon atom of any N-nitrosamines by cytochrome P450, predominantly in the liver by CYP2E1 (Bellec et al., 1996) and other isoforms (Yamazaki et al., 1992; Chowdhury et al., 2012) In this work we only consider compounds hydroxylated at the α-carbon atoms, though there are also known cases for β- or γ-hydroxylation (Snodin et al., 2024). A detailed quantum chemical assessment of the reaction mechanism for Cyp-mediated hydroxylations of four nitrosamines including **NDMA** and **NPYR** and via stepwise high-spin quartet or concerted low-spin doublet states have been described by the Schüürmann group (Ji and Schuurmann, 2012; Ma et al., 2018). Since this first step is enzyme-catalyzed with two competing mechanisms, but always strongly exothermic as shown by Wenzel et al. (2022), and the actual reactive position determined by electronic and steric effects, it is not considered in our work. And there is a concurrent deactivation step of denitrosation via the step-wise mechanism described by Schüürmann.

The first step with transition state **1-2_TS** considered in this work is the proton transfer from the α-hydroxylated product **1** to the N-nitrosamines e oxygen in **2**. For aliphatic N-nitrosamines this results in elimination of an aldehyde, and for cyclic systems in ring-opening. For non-symmetric molecules, α-hydroxylation occurs preferably on the smaller, less sterically hindered and less electron-rich α-carbon atom.

The second step **2-3** is the barrierless generation of the diazonium cation **3** via catalytic elimination of the hydroxyl group. Cyclic aliphatic moieties can then further undergo intramolecular ring closure via reaction with the terminal aldehyde group, (Dobo et al., 2022; Li and Hecht, 2022), not altering their DNA-reactivity.

**FIGURE 2:**
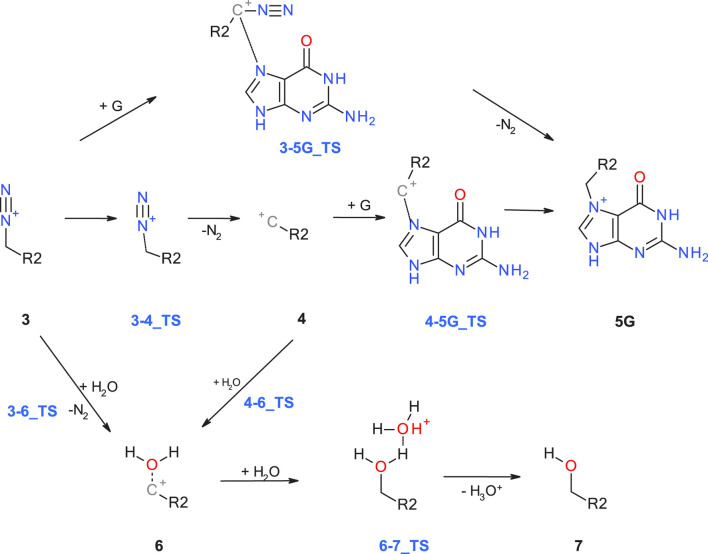
Alternate pathways for the reaction of diazonium ion **3** with either DNA base (G: guanine) or water.

Starting with the diazonium ion **3**, a multitude of potential follow-up steps have to be considered, as mapped out in Figure 2. The first option is the direct DNA-alkylation of the base by aliphatic or aromatic diazonium ions via S_N_2/S_N_Ar reactions to adduct **5G** via transition state **3-5G_TS**, with elimination of N_2_. This is considered the preferred reaction for all cases where the corresponding carbenium ions are instable. Alternative DNA adducts in case of aryl-N-nitrosamines (not relevant for the N-nitrosamines discussed here) are C8 adenine and guanine adducts and azo C8-adducts (cf. Figure 4 of Wenzel et al. and references therein).

The second option is deactivation of the diazonium ion **3** via hydrolysis yielding a water adduct **6** that finally results in the respective alcohol **7**. The reaction for aliphatic and aromatic diazonium ions proceeds predominantly via S_N_2 or S_N_Ar mechanisms with transition state **3–6_TS** with water as partner (Dipple, 1995; Wu and Glaser, 2004).

Elimination of N_2_ results in the respective carbenium ion intermediate **4** that again can form either DNA adduct **5G** (option 3) or water adduct **6** (option 4). Both are competing S_N_1 reactions of the carbenium ion.

## Results

### DNA base reactivity

**FIGURE 3:**
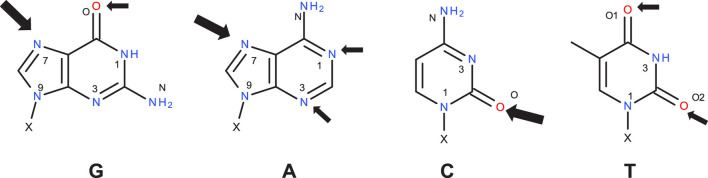
Structures of the DNA bases guanine (G), adenine (A), cytosine (C), and thymine (T), with experimentally observed positions for nucleophilic attack [cf. (Wenzel et al., 2022)] resulting in potential alkylations marked by thick and thin arrows for prominent and less likely positions. X denotes the bond to the DNA backbone. Table 2 lists the respective reaction free energies for reactions with a phenyl cation.

DNA alkylation can occur at different nucleophilic positions, as reported by experimental as well as computational approaches^,^ (Dipple, 1995; Bhattacharjee, 2014), and shown in Figure 3. Since we would like to focus on a model reaction with one specific nucleobase for the activation profiles in this work, we first calculate the reaction free energies of DNA base with an phenyl cation (in line with Wenzel et al. to enable comparison) for the various positions in the four DNA bases, as given in Table 2, which are calculated intrinsic reactivities ignoring steric accessibilities and the energies needed to break the base pair interactions.

**TABLE 2:** Intrinsic reaction free energies in kcal/mol for the reactions of the DNA bases with a phenyl cation. Calculations were done with M06-2X-D3(BJ)/aug-cc-pVTZ//B3LYP-D3(BJ)/6-311+G (d,p) in SMD solvent model and water as solvent.

Guanine		Adenine		Cytosine		Thymine	
position	ΔG_react_	position	ΔG_react_	position	ΔG_react_	position	ΔG_react_
7	−72.89	7	−70.76	O	−62.76	O1	−53.06
3	−69.90	3	−71.86	1	−38.19	3	−29.83
1	−69.49	1	−73.36	3	−73.60	O2	−50.49
O	−60.89	N	−55.47	N	−52.92	1	−32.88

For **G** the reported most prominent reactive position (Dipple, 1995) is also the one with the highest ΔG_react_ with −72.9 kcal/mol, whereas for **A** the minor site 1 is more reactive than site 7 by 1.6 kcal/mol. For both bases, positions 7 are not involved into base pairing, but the bases are connected to the backbone via 9-position. On the other hand, in **C** both the intrinsically more reactive position 3 as well as the oxygen are involved in pairing. For **T**, both oxygens are significantly more reactive than the nitrogens, as reported before. Only O2 is involved in pairing.

As a side note, as expected for tautomeric positions, the intrinsic reaction free energies for the not accessible positions 9 in **G** and **A** that are connected to the backbone are very similar to the values for the positions 7, with −72.3 and −71.2 kcal/mol for **G** and **A**, respectively.

### Carcinogenic N-nitrosamines NDMA and NPIP

In this first section we discuss the activation profiles of two carcinogenic compounds from the cohort of concern N-nitrosamines, namely, the highly potent (AI = 96 ng/d) N-nitroso dimethylamine (**NDMA**, AC01 in Wenzel et al.) and less potent (AI = 1,300 ng/d) N-nitrosopiperidine (**NPIP**, CYC06). The aim is twofold: We want to identify if the change of substituent from open chain to aliphatic ring and the accompanying change from a leaving aldehyde to an aldehyde being part of the reactive species will result in changes of the electronic effects on the diazonium ion **3** or the carbenium ion **4**. Secondly, the two compounds allow to compare for consistency with the results of Wenzel et al. obtained with our slightly adapted calculation setup.

**FIGURE 4:**
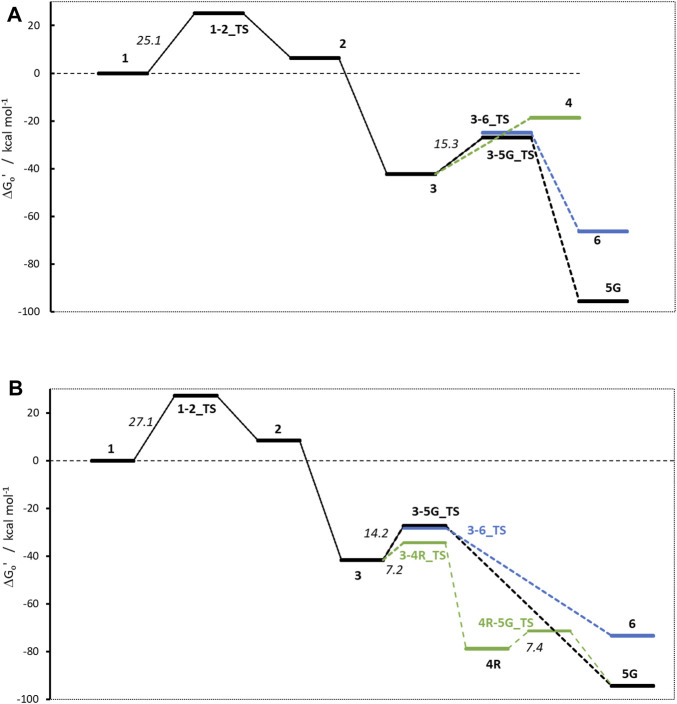
Activation profiles for **(A)** NMDA and **(B)** NPIP. For this and all following profiles, the relative free energy of α-hydroxylated N-nitrosamines (1) is set to 0 kcal/mol. To enhance readability, carbenium ion pathways are indicated in green, water adducts are depicted in blue.

Figure 4A shows the activation profile for **NMDA** and Figure 4B the profile for **NPIP**. As reported by Wenzel et al. (2022) and confirmed by our calculations, the initiation step of α-hydroxylation is enzyme-catalyzed and always strongly exothermic for N-nitrosamines, thus not rate-determining. It is omitted in our analysis throughout. By using a cytochrome P450 model system, the reaction free energies for all molecules in the previous work (Wenzel et al., 2022) were between −86.9 and −91.8 kcal/mol, whereas our values (see Table 3) obtained from the simpler model reaction with dioxygen are between −136.1 and −140.3 kcal/mol, i.e., just shifted by about 50 kcal/mol.

**TABLE 3:** Reaction free energies and activation free energies for the nine N-nitrosamines. All values are in kcal/mol and relative to the hydroxylated nitrosamine intermediates **1** that were set consistently to 0 kcal/mol. Reaction free energies are obtained in a way to conserve the numbers of atoms and as much as possible the types of bonds. Numbers in brackets are the transition state barriers relative to the reactant. Cases without identified transition state are indicated by a dash.

	NDMA *strongly positive*	NPIP *positive*	NTBA *negative*	BBNA *negative*	NPZ *positive*	NMA *positive*	2-NMPY *positive*	3-NMPY *negative*	4-NMPY *negative*
0	+136.1	+89.3	+136.0	+137.2	+140.3	+139.1	+139.4	+139.0	+139.8
1	0.0	0.0	0.0	0.0	0.0	0.0	0.0	0.0	0.0
1-2_TS	+25.1	+27.1	+23.9	+14.3	+28.6	+26.4	+26.4	+26.6	+27.5
2	+6.3	+8.5	+10.4	−14.7	+9.0	+6.0	+6.5	+6.2	+6.7
3	−42.3	−41.6	- ^b^	−65.3	−40.2	−36.3	−30.8	−32.5	−28.5
3-4_TS	-	−34.4 (+7.2)	-	−64.6 (+0.7)	−32.8 (+7.5)	-	-	-	-
4	−18.6	−78.8 ^a^	−74.4	−82.5 [-76.6] ^a^	−101.3	−20.3	−32.2	−15.0	−16.4
4-5G_TS	-	−71.4 (+7.4)	-	-	−87.5 (+13.8)	-	-	-	-
3-5G_TS	−27.0 (+15.3)	−27.4 (+14.2)	-	−57.0 (+8.3)	−28.8 (+11.4)	−3.5 (+32.8)	−16.0 (+14.7)	−0.5 (+32.0)	+3.5 (+25.0)
5G	−95.5	−94.3	−95.7	−116.0	−93.3	−93.2	−93.1	−92.3	−91.5
3-6_TS	−25.0 (+17.4)	−28.2 (+13.4)	-	-	−25.3 (+11.4)	-	-	-	-
6	−66.4	−73.4	−76.3	−92.3	−87.8	−64.5	−66.9	−61.8	−63.1

^a^ cyclic carbenium ion;

^b^ Diazonium ion not existent due to high stability of carbenium ion.

The first linear step (see Figure 1) after hydroxylation is the proton transfer from the hydroxy group of **1** to the nitrosamine group of **2** via transition state **1–2_TS** (Bond lengths and angles for all transition state structures are given in Supplementary Tables T1, T2 and depictions of the definitions of the respective values in Supplementary Figure F1). Though the two reactions for **NDMA** and **NPIP** result in different types of reaction products, i.e., in the case of **NDMA** formaldehyde is released and in the case of **NPIP** the aliphatic aldehyde is part of the hydroxy nitrosamines species, the reactions have similar activation free energies of 25.1 (24.3) and 27.1 (20.8) kcal/mol (values from Wenzel et al. in brackets) and reaction free energies of +6.3 (+8.2) and +8.5 (+5.2) kcal/mol respectively, relative to the hydroxylated nitrosamines that was set to zero kcal/mol. As reported, (Wenzel et al., 2022), the ring opening, depending on ring size, is exothermic for smaller, strained rings, endothermic for 5- to 7-membered rings, and again exothermic for larger ones.

The second linear activation step is highly exothermic and proceeds without activation barrier. It is the acid-catalyzed formation of the diazonium cation **3** by elimination of the hydroxy group from **2**. Again, reaction free energies are comparable with −42.33 (−42.6) and −41.6 (38.9) kcal/mol for **NDMA** and **NPIP**.

As shown in Figure 2, starting with the diazonium cation **3,** multiple competing pathways can be pursued, leading either to DNA adducts or to deactivation of the N-nitrosamine. The ratios of DNA adducts **5G** and water adducts **6** are determined by three contributions: thermodynamic stabilities of the relative free energies of the products, kinetic contributions due to transition state (TS) barriers, and as a third major factor, the balance between the diffusion-controlled reaction with DNA bases and the reaction with excess water molecules, which is statistically by orders of magnitude more likely.

The first DNA activation pathway is via an S_N_2 reaction of the diazonium ion **3** with DNA base, considered to be the usual pathway (Wenzel et al., 2022). As mentioned before, the model reaction reported here is the one with N7 of guanine **G**. Reactions to the DNA adducts **5G** are strongly exothermic for both molecules with −95.5 and −94.3 (−91.4) kcal/mol and TS barriers of +15.3 (+15.1) and +14.2 (+11.5) kcal/mol for **NDMA** and **NPIP**. Both the reaction energies and the barriers are comparable for the two molecules, as for the larger congeneric series discussed in the earlier publication (Wenzel et al., 2022).

**FIGURE 5:**
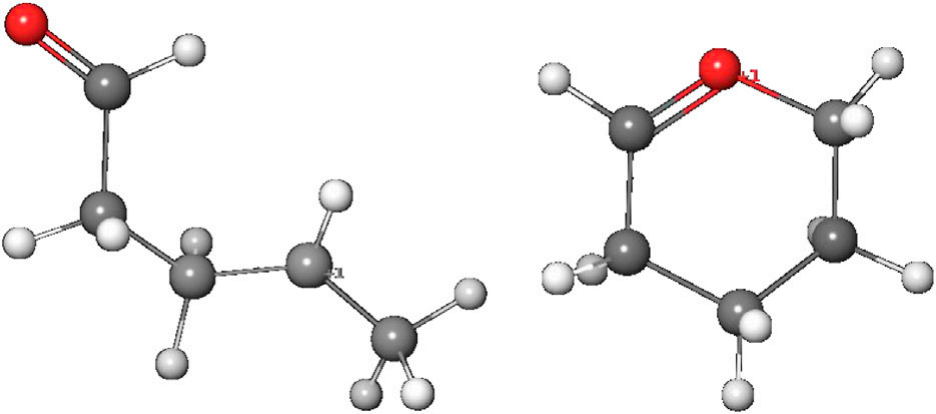
Open-chain and cyclic NPIP carbenium ions **4** and **4R**.

The alternate activation pathway proceeds via S_N_1 reaction of the intermediate carbenium ion **4** retained from dinitrogen cleavage. Here we see a significant difference in the activation profiles of the two molecules. Whereas in the case of **NDMA** the carbenium ion **4** is higly unstable with +23.7 kcal/mol relative to **3**, in case of **NPIP** the reaction is strongly exothermic with −37.2 (−5.2) kcal/mol and a TS of only +7.2 kcal/mol, in contrast to the results of Wenzel et al. The reason for the contradictory reaction profiles is in the fact that we identify a cyclic carbenium ion **4R** that is considerably more stable by −20.7 kcal/mol than the open-chain carbenium ion **4** (Figure 5).

For **NDMA**, no TS barrier for N_2_ cleavage could be identified by reaction coordinate scans.

Deactivation of diazonium ions **3** to the water adducts **6** for both molecules proceed via transition states **3–6_TS**. The TS barriers for **NDMA** and **NPIP** are +17.4 and +13.4 (+9.4) kcal/mol and the reaction free energies −66.4 and −73.4 (−66.1) kcal/mol, again comparable between the two molecules.

Finally, the carbenium ion **4R** in case of **NPIP** can react with either DNA or water to form the respective adducts. We find a low barrier **4R-5_TS** of +7.4 kcal/mol but no barrier for the reaction with water. Since Wenzel et al. (2022) did not identify the stabilized cyclic **4R**, they state that there would be no transition states for the two competing reactions. For **NDMA**, carbenium ion **4** is too unstable to be a likely intermediate.

Comparing the two profiles, we find that in case of **NDMA** the reactions proceed solely via diazonium ion **3** to the DNA and water adducts, via very similar transition state barriers. Since the DNA adducts are more stable, the product ratio is thermodynamically controlled. In the case of **NPIP**, the reaction will mostly proceed via the cyclic carbenium ion **4R**. There is a low TS barrier to the DNA adduct but no barrier to the water adduct. The water adduct is higher in energy than the DNA adduct, but about identical in energy to the DNA adduct TS. The ratio for **NPIP** adducts will thus be mostly controlled by the availability of the reactants DNA base and water being available in excess, thus statistically controlled.

### Non-carcinogenic N-nitrosamines NTBA and BBNA

The stability of the carbenium ion **4** is the only major difference in the two profiles of **NDMA** and **NPIP**. Therefore, we selected two non-carcinogenic compounds, namely t-butyl(methyl) (nitros) amine (**NTBA**) and bis(butan-2-yl) (nitros)amine (**BBNA**), that are suspected to comprise even more stabilized carbenium ions **4**.

**FIGURE 6:**
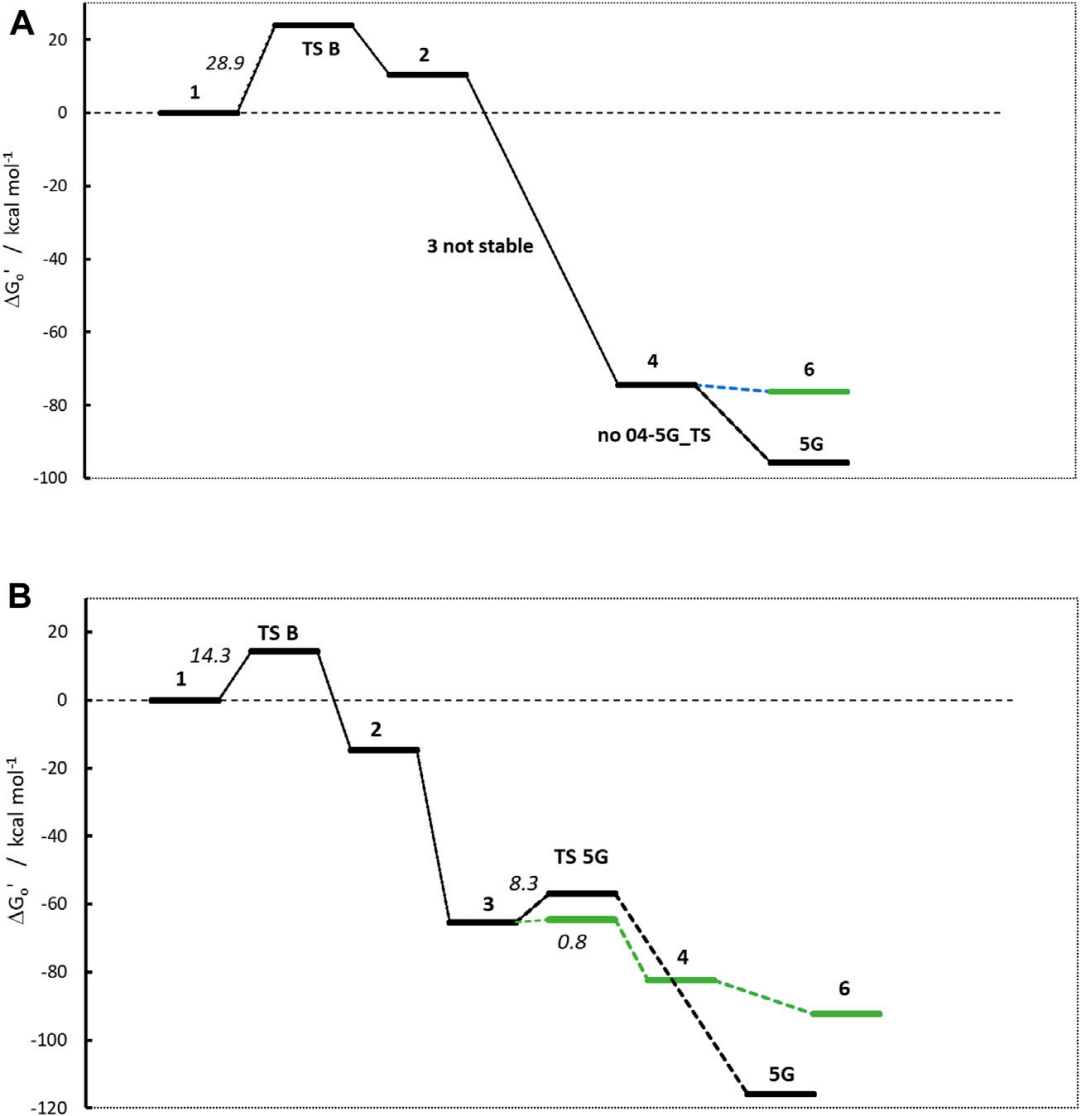
Activation profiles of **(A)** N-nitrosomethyl-tert-butylamine (NTBA) and **(B)** bis(butan-2-yl) (nitros)amine (BBNA).

Obviously, the profiles in Figure 6 look different from the ones in Figure 4. For **NTBA**, the release of formaldehyde upon proton transfer from the hydroxy group of **1** to the nitrosamine group of **2** via transition state **1-2_TS** is again endothermic by +10.4 kcal/mol with a barrier of +23.87 kcal/mol. Contrary, for **BBNA**, butylaldehyde is cleaved, and the reaction is exothermic by −14.7 kcal/mol with a lower TS of +14.3 kcal/mol.

The second linear activation step is again highly exothermic for both compounds and proceeds without TS barrier. The diazonium ion **3** for **BBNA** is, due to the positive inductive effect of the aliphatic chain, significantly more stable than for **NDMA** or **NPIP** with −65.3 kcal/mol (compared to −42.3 and −38.9 kcal/mol). **NTBA** even directly reacts to the carbenium ion **4** (−74.4 kcal/mol). Multiple attempts to identify diazonium ion **3** were not successful, and its existence can be ruled out. The N_2_ cleavage from **3** to carbenium ion **4** in the **BBNA** pathway is exothermic by −17.2 kcal with a negligible barrier of +0.8 kcal/mol.

The **NTBA** derived t-butyl cation **4** readily forms both the DNA adduct **5G** (−95.7) and the water adduct **6** (−76.3 kcal/mol). The analogous reaction free energies for **BBNA** have values of −116.0 and −92.3 kcal/mol for DNA and water adducts, respectively. Both reactions for both compounds are barrierless. Therefore, one would expect creation of the DNA adduct to proceed mostly via the barrierless reaction **4** to **5G** and less by the reaction of **3** to **5G** that has a TS barrier. Though DNA adducts **5G** for both compounds are more stable by about 20 kcal/mol, one can always expect a certain preference of the water adduct, since the reaction with DNA is diffusion-controlled whereas the one with water not, with water being available in excess. The water adducts than finally react to the respective t-butyl and i-butyl alcohols.

### Carcinogenic NPZ

N-nitrosopiperazine **NPZ** is the piperazine analog to the piperidine **NPIP**, i.e., has a nitrogen in the para position as compared of the aliphatic carbon in the latter. Although both molecules differ only by one atom, carcinogenicity data reveal a significant difference in potency as reflected by the derived AIs of 28,500 (**NPZ**) and 1,300 ng/day (**NPIP**) (Bercu et al., 2023).

**FIGURE 7:**
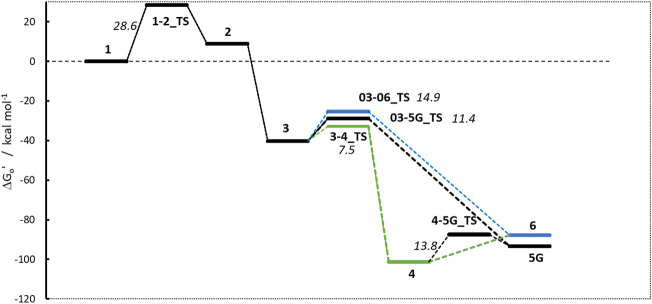
Activation profile of N-nitrosopiperazine (NPZ).

The linear part of the activation profile for **NPZ** in Figure 7 compares well to the one of **NPIP** (values in brackets). Proton transfer from **1** to **2** proceeds via a TS barrier **1-2_TS** (as seen for all compounds) of +28.6 kcal/mol (+27.1) and is endothermic by +9.0 kcal/mol (+8.5). The reaction to diazonium ion **3** is again barrierless and exothermic by −40.24 kcal/mol (−41.6) compared to **1**.

**FIGURE 8:**
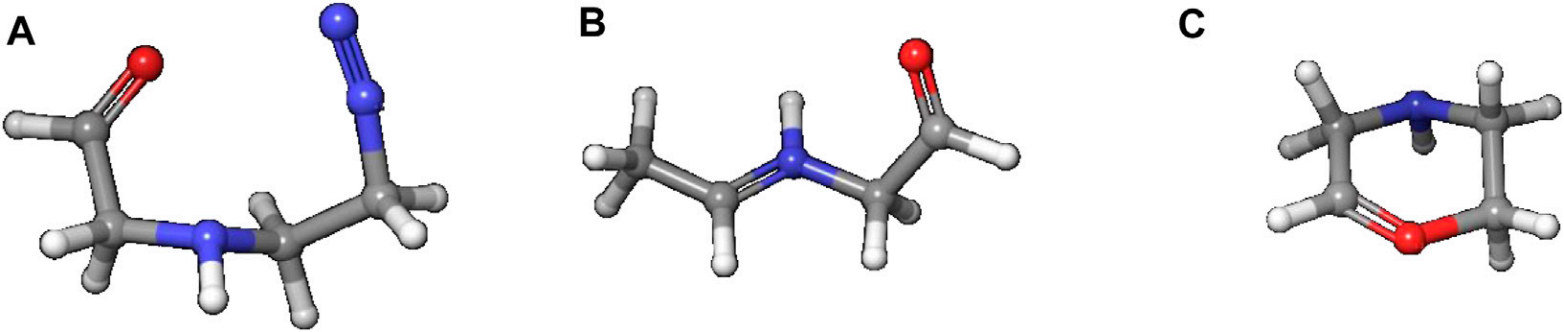
Structures of diazonium ion **3 (A)** and the two cationic species in of the activation pathway for N-nitrosopiperazine (NPZ), namely, nitrenium ion **4** **(B)**resulting of proton shift and **(C)** a cyclic oxonium ion **4R**.

Reaction to cation **4** proceeds via transition state **3–4_TS** with an activation barrier of +7.5 kcal (+7.2) and reaction free energy of −101.3 kcal/mol (−71.4). The resulting stabilized cation is nevertheless not a carbenium ion, but the more stable nitrenium ion obtained from proton shift from the β-to the α-carbon after N_2_ release, as can be seen by comparing diazonium ion **3** in Figure 8A and nitrenium ion **4** in Figure 8B. Several attempts to derive the structure of the expected carbenium ion were not successful, proving the strong driving force of charge stabilization. But we identified an alternate ring closed oxonium ion **4R** (Figure 8C) that is less stable than **4** by 24.7 kcal/mol. In contrast, for **NPIP** the cyclic form was more stable than the open form, indicating the major effect of the carbon nitrogen replacement.

The transition states for the S_N_2 reactions from **3** to **5G** of +11.4 kcal/mol (+14.2), from **3** to **6** of +14.9 (+13.4) are again similar and also similar to the ones of **NPIP** (values in brackets). The same is true for the lower barriers from **3** to **4** of +7.5 kcal/mol (+7.2). The main distinction is the structure and free energy of the carbenium ion **4**. For **NPZ** it is open-chain with charge localization mostly on nitrogen and for **NPIP** it is cyclic with charge localization more on oxygen. The **NPZ** ion **4** is significantly more stable with −101.3 kcal/mol than the **NPIP** derived **4** (−78.8). In addition, the transition state barrier **4-5G_TS** for **NPZ** is higher than for **NPIP** with values of 13.8 and 7.4 kcal/mol. In both cases no barrier for the reaction to the water adduct **6** was identified.

Taking all the data into account, the higher stability of carbenium ion **4** and the higher barrier **4-5G_TS** make **NPZ** less amenable to **DNA** adduct creation than **NPIP,** in accordance with the experimental results from Bercu et al. (2023).

### N-nitrosamines with aromatic substituents

The last set of molecules discussed here is a congeneric series of N-nitrosamines with one methyl substituent as in **NDMA** and **NTBA**, and the second substituent being an aromatic ring, either phenyl or 2-, 3- or 4-pyridyl. Whereas the phenyl compounds N-nitrosomethylaniline **NMA** (Boyland et al., 1964) and **2-NMPY** (Preussmann et al., 1979; Schmahl, 1975) were reported to be carcinogenic, **3-NMPY** and **4-NMPY** (Preussmann et al., 1979; Schmahl, 1975) are non-carcinogenic, based on existing rat *in vivo* studies.

It is well-known that the phenyl cation is extremely unstable (Breton, 2018) due to the fact that the positive charge is located in the pi-system, thus revoking the aromatic character of the ring system. It can only be generated in gas-phase (Patzer et al., 2010) or trapped in argon matrix at 10 K (Winkler and Sander, 2006). The low cation stability is especially obvious when comparing to cyclohexyl and even more t-butyl cations. We expected this to be visible in the activation profiles that should look more similar to the ones of **NDMA** and **NPIP**. We also expected distinct differences in the profiles of the phenyl and the three pyridyl compounds due to different nitrogen positions in the ring systems that should influence the overall reactivity and thus the profiles.

**FIGURE 9:**
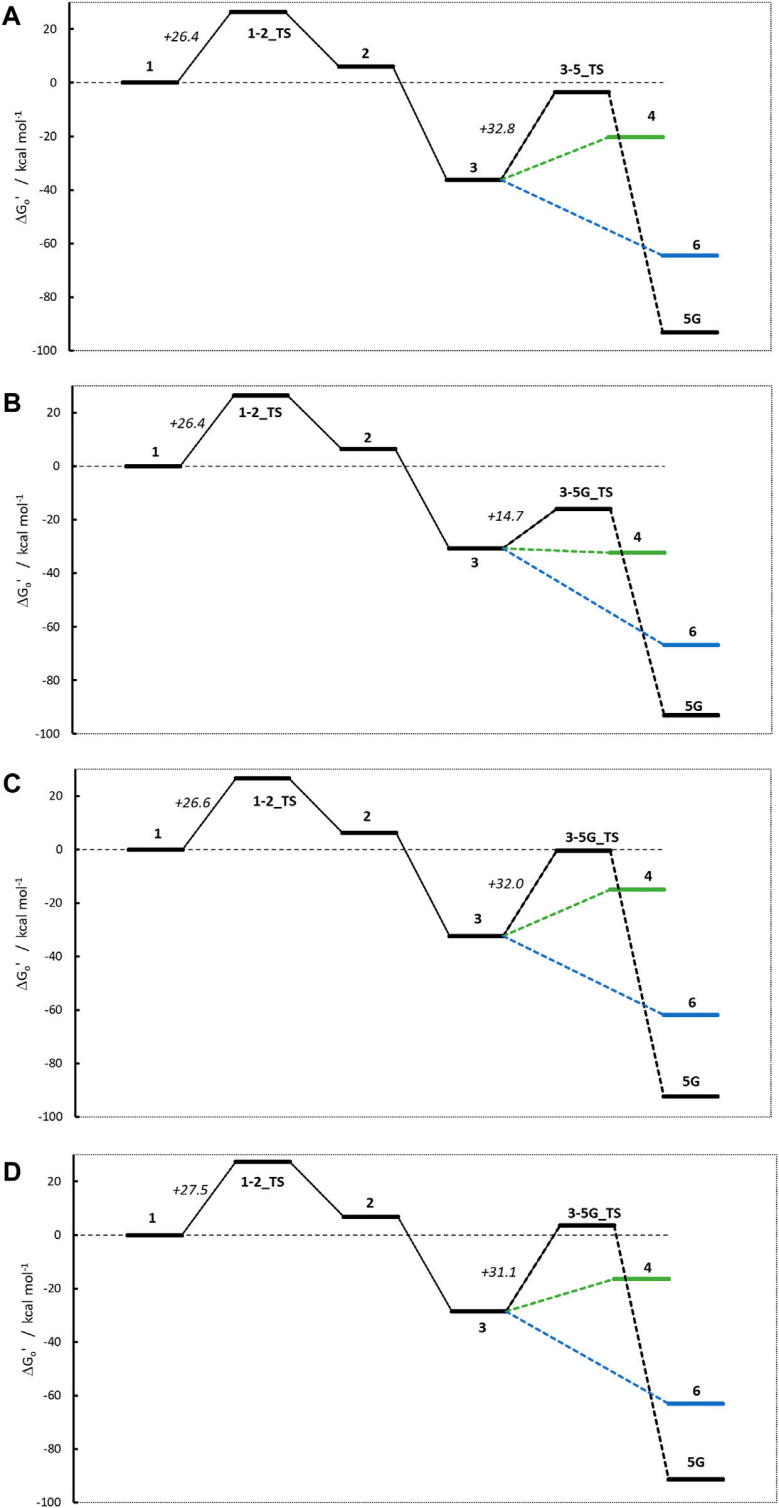
Activation profiles for **(A)** N-nitroso-N-methylaniline (NMA), **(B)** N-nitroso-N-methyl-2-aminopyridine (2-NMPY), **(C)** N-nitroso-N-methyl-3-aminopyridine (3-NMPY) and **(D)** N-nitroso-N-methyl-4-aminopyridine (4-NMPY).

Figure 9 shows the four activation profiles, and the respective reaction free energies and transition state barriers are reported in Table 3. As anticipated, they resemble the profiles seen for **NMDA** and **NPIP**, but contrary to our expectations and to the toxicological data, the four profiles are almost indistinguishable, except for the free energies of cations **4** and the missing transition states **3–6_TS**.

The first step, the proton transfer from **1** to **2**, is endothermic for each of the four compounds by about 6.0 kcal/mol (**NMA**: +6.0; **2-NMPY**: +6.5; **3-NMPY**: +6.2**; 4-NMPY**: +6.7 kcal/mol) with activation barriers of +26.4, +26.4, +26.6, and +26.6 kcal/mol. The values for step 1 are thus almost identical for all molecules. Reactions to the diazonium ions **3** again are barrierless and strongly exothermic with −36.3 kcal/mol for **NMA**, −30.8 for **2-NMPY**, −32.5 for **3-NMPY** and −28.5 for **4-NMPY**. The slightly lower free energies of the pyridine diazonium ions compared to the phenyl diazonium ion can be explained with the electron-deficient nature of the aromatic rings.

The diazonium ions **3** readily react without transition state to the water adducts **6** with free energies of −64.5 (**NMA**), −66.9 (**2-NMPY**), −61.8 (**3-NMPY**) and −63.1 kcal/mol (**4-NMPY**). The respective reactions to the DNA adducts **5G** (**NMA**: −91.2; **2-NMPY**: −93.1; **3-NMPY**: −92.3; **4-NMPY**: −91.5 kcal/mol) proceed via transition states with barrier heights of +32.8 (**NMA**), +14.7 (**2-NMPY**), +32.0 (**3-NMPY**) and +25.0 kcal/mol (**4-NMPY**). Thus, the water adducts **6** and DNA adducts **5G** are equally stable for the four compounds, but the barrier **3-5G_TS** for **2-NMPY** is considerably lower than for the others. The DNA adducts **5G** are consistently more stable than the water adducts **6** as seen for all compounds in this work, but the reactions are kinetically hindered (proceeding via transition states) and diffusion-controlled, leading to overall reduced mutagenic/carcinogenic potential, compared to **NDMA** and **NPIP** with similar barrier heights for the activation and the deactivation reactions. The alternate dinitrogen cleavage to the resulting carbenium ions **4** is not likely due to the instability of the respective cations, though one can calculate the respective structures which have free energies about 15 kcal/mol higher than diazonium ions **3**. The one exception here is the 2-pyridium cation **4** that is about isoenergetic with the respective diazonium ion **3** since it is stabilized by hyperconjugation of the nitrogen lone pair into the empty orbital of the positively charged carbon atom. As shown by Breton et al. (2023) using natural bond orbital analysis, (Glendening et al., 2013), both the positive carbon and the nitrogen are sp-hybridized and form an in-plane π-bond, by this resembling an electronic configuration like benzyne. The same type of stabilization explains the lower barrier **3-5G_TS** in case of **2-NMPY**.

Comparison of the four profiles in Figure 9 does not reveal significant differences that would explain the reported carcinogenicity of **NMA** and **2-NMPY** and its absence in case of the other two compounds. The only differentiation in the series is the lower barrier **3-5G_TS** for **2-NMPY** and the more stable carbenium ion **4,** but that would not explain the carcinogenicity of **NMA**. A thorough investigation of relevant literature cited in the original works provides a hint that the cause for the experimental observations might indeed not be the activation profile but the metabolic routes.

**FIGURE 10:**
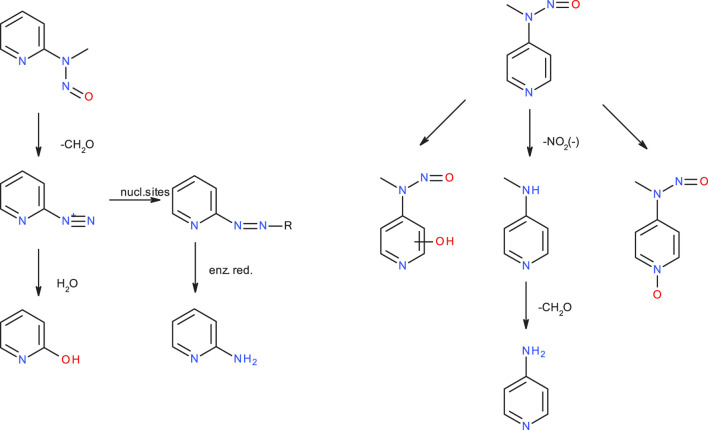
Major metabolic pathways for N-nitroso-N-methyl-2-aminopyridine (**2-NMPY**, left) and N-nitroso-N-methyl-2-aminopyridine (**4-NMPY**, right) from *in vivo* study described in text.

Kroeger-Koepke et al. (1983) reported that there was no apparent correlation between mutagenicity and the rates of N-demethylation for several **NMA** derivatives leading to the formation of the respective benzene diazonium ions, that **NMA** and p-F-NMA were readily demethylated by rat liver S9, and that this appeared to be the principal *in vitro* metabolic route and also likely to be the important pathway *in vivo*. (Michejda et al., 1982). Nevertheless, though **NMA**, p-F-NMA and p-NO_2_-NMA are readily metabolized, p-F-NMA was shown to be less carcinogenic than p-NO_2_-NMA and not to induce tumors (Kroeger-Koepke, 1983). In the publication on the carcinogenicity of the three **NMPY** derivatives, Preussmann et al. (1979) mention that 3-diazopyridine preferably forms diazo compounds via coupling reactions, whereas the 2- and 4-diazopyridines decompose quickly, but the authors admit that the observed carcinogenicity of **2-NMPY** and the absence in case of the other derivatives cannot be explained by chemical reactivity alone. In a follow-up *in vivo* study collecting rat urine, the metabolites of **2-NMPY** and **4-NMPY** were identified (see Figure 10), whereas the metabolites of **3-NMPY** could not be sufficiently detected due to low amounts administered based on the high acute toxicity of the compound. (Heydt-Zapf et al., 1983). After 48 h no unchanged N-nitrosamines were detected for **2-NMPY**, but 2-hydroxypyridine, the hydrolysis product of putative diazonium intermediate, as well as 2-amino-pyridine, expected to stem from conjugates with glucuronic acids and sulfates were found instead. In case of **4-NMPY**, apart from 1% unchanged compound, a mixture of the N-oxide, denitrosated and ring-hydroxylated products were found, but no 4-hydroxypyridine which would be indicative of the diazonium ion.

Taking all the experimental and computational facts together, we see that an activation profile favoring DNA adduct formation is a *conditio sine qua non*, but not sufficient. Instead, the metabolic activation step which is not amenable to a computational study on reactivity, determines if the diazonium ion will be formed at all. For the four compounds discussed here, as said, we have an incomplete picture, because not all data needed are available. Computed activation profiles for the four compounds strongly hint towards DNA adduct formation, however with competing deactivation. This results in lower carcinogenic potency compared to **NDMA** or **NPIP**. Experimental data show that **NMA** is readily demethylated, i.e., it is likely that the diazonium ion is formed, but no metabolite study exists. In case of **2-NMPY** the observed metabolite 2-hydroxypyridine and the absence of 4-hydroxypyridine for **4-NMPY** explain the observed carcinogenicity. Finally, there is no data at all for **3-NMPY** to complete the picture. These findings suggest that a “false positive” result can be obtained from applying quantum chemical calculations if the hypothesis of successful metabolic activation does not hold true for a given nitrosamine. Crucially though, we see no scenario under which “false negatives” could occur, making the presented method a rather conservative approach.

### Summary of findings

**FIGURE 11:**
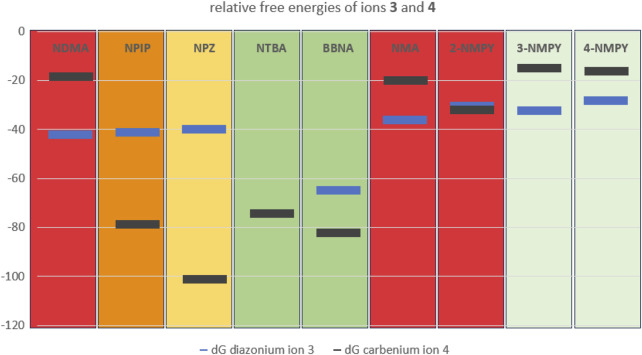
Plot of the free energies in kcal/mol of diazonium ion **3** (blue) and carbenium ion **4** (dark grey) relative to starting structure **1**.Background color-coding is representative of carcinogenic potential of the substances (for details cf. Table 1). Note that no diazonium ion exists for **NTBA** and that **3-NMPY** and **4-NMPY** are negative due to different activation pathways, as indicated by lighter green background.

From the nine profiles we have calculated, we can deduce that the relative free energies of the ions **3** and **4** to starting structure **1** and even more the relative stabilities of the two ions determine the tendency to form DNA adducts that than can cause mutagenicity and carcinogenicity. Excluding **3-NMPY** and **4-NMPY** for the reasons provided before, we find that the more stable the carbenium ion **4** the higher the AI values. The examples of **NTBA** and **BBNA** show that in case the stable carbenium ion **4** coexists with a stable or even transient diazonium ion **3**, the compounds are non-cancerogenic. This is intuitively visible from Figure 11 that compares the relative free energies of the nine compounds.

## Discussion and outlook

This work presents a computational method applicable to N-nitrosamine impurities and NDSRIs (or representative fragments) requiring evaluation in terms of their mutagenicity and carcinogenic potency. Specifically, for molecules where CYP activation cannot be excluded, quantum chemical calculations can provide valuable insights (e.g., as part of a weight of evidence approach) into the balance between potential formation of a DNA adduct and the competing deactivation reaction to the water adduct and the resulting alcohol. Using examples with *in vivo* carcinogenicity data, we demonstrated that reaction profiles of cohort of concern substances such as **NDMA** differ significantly from the profiles of substances considered non-carcinogenic. Our approach can be considered complementary to the recently published (EMA, 2022) carcinogenic potency categorization approach providing an additional way to estimate carcinogenic potency based on structural motifs together with unprecedented mechanistic insights into their reactivity. The value of such methods is further underlined by the challenge of analytically detecting N-nitrosamine concentrations significantly below the TTC (Schlingemann et al., 2023).

Our quantum chemical calculations show that the first three linear steps in the reaction cascade after nitrosation of the amine groups, for all compounds considered by us and by others, have very similar activation profiles. The cytochrome P450-catalyzed hydroxylation of the aliphatic α-carbon is strongly exothermic, followed by slightly endothermic hydrogen shift to the NO-group with transition state barriers of around 25 kcal/mol, and barrierless exothermic reaction to the diazonium ion.

For the second, non-linear part of the profile, we find that the relative stabilities of the diazonium ion and carbenium ion are decisive for the balance between activation and deactivation. In case of unstable carbenium ions we find predominantly S_N_2 reactions of the diazonium ion with DNA and water adducts, in case of stabilized carbenium ions we expect the reactions predominantly to proceed via the carbenium ions which barrierless (with the exception of **NPIP** and **NPZ**) can form the DNA adduct and the water adduct. For the cases with energetically similar DNA and water adducts the water adduct is expected to be preferred due to water being available in excess.

This is especially exemplified by the series of **NDMA**, **NPIP,** and **NPZ**. Whereas for **NDMA** the methyl carbenium ion is unstable and the reactions of the diazonium ion with DNA and water proceed via transition state barriers of similar heights, i.e., mainly kinetically controlled, for **NPIP** there is only a low barrier to significantly more stable carbenium ion that can barrierless react to the water adduct, but higher barriers for the kinetically controlled reactions to DNA and water adducts. For **NPZ** we find that the cation is further stabilized by charge localization on the nitrogen, i.e., a formal nitrenium ion, which is lower in energy than the DNA adduct, and the water adduct. Both DNA adduct formation from diazonium ion and from nitrenium ion proceed kinetically controlled, whereas the reaction to the water adduct is barrierless. The profiles for the three compounds fit well to the experimentally observed carcinogenicity for the three compounds leading to acceptable intakes of 145 ng/d for **NDMA**, 1,300 ng/d for **NPIP** and 28,500 ng/d for **NPZ**, as shown in Figure 11 The strong correlation between AIs and carbenium ion free energies that we find for the three examples nevertheless has to be confirmed by further calculations on more examples.

Finally, based on the calculations for the congeneric series of the aryl N-nitrosamines, we conclude that the calculation of the reaction profiles can provide an indication of the maximal risk of DNA adduct formation. In this series, the initial metabolic activation that is not covered by our work is decisive for the observed carcinogenicity, though from the profiles all compounds would be considered to show similar carcinogenicity.

Initially, the topic of nitrosamine formation in drug substances and drug products was limited to small nitrosamines for which animal carcinogenicity data are often available. In the recent years however, NDSRIs became an additional challenge due to lack of such robust data and low resemblance to the small nitrosamine impurities (e.g., making read-across approaches unfeasible). To ensure patient safety while at the same time keeping pharmaceutical products available, strategies to assess the carcinogenic potency of NDSRIs, such as the recently published structure activity relationship based CPCA, are required (Kruhlak et al., 2024). Our investigations show that quantum chemical calculations can be a method to refine certain aspects of the CPCA (e.g., distinguishing molecules lacking any carcinogenic potential) and increase its underlying knowledge base.

## Data Availability

The original contributions presented in the study are included in the article/Supplementary Material, further inquiries can be directed to the corresponding author.

## Abbreviations

AI, acceptable intake; API, active pharmaceutical ingredient; BBNA: bis(butan-2-yl) (nitros)amine; CYP, cytochrome P450; ICH, International Council for Harmonisation of Technical Requirements for Pharmaceuticals for Human Use; DS, drug substance, DP, drug product; HA, health authorities; NDMA, N-nitrosodimethylamine; NDEA, N-nitrosodiethylamine; NDSRI, nitrosamine drug substance related impurity; NMA, N-nitroso-N-methylaniline; 2-NMPY: N-nitroso-N-methyl-2-aminopyridine; 3-NMPY: N-nitroso-N-methyl-3-aminopyridine; 4-NMPY: N-nitroso-N-methyl-4-aminopyridine; NPIP: N-nitrosopiperidine; NPZ, N-nitrosopiperazine; NTBA, N-nitrosomethyl-tert-butylamine, TD50, tumor dose 50%; TTC, threshold of toxicological concern; TS, transition state.
