# Supplementary material for: Quantum chemical calculations of nitrosamine activation and deactivation pathways for carcinogenicity risk assessment
Source: Front Pharmacol. 2024 Jul 17;15:1415266. doi: 10.3389/fphar.2024.1415266 (PMC11288830; doi:10.3389/fphar.2024.1415266)
Supplement: Supplementary file 1 [file Table1.DOCX]

Supplementary Information

Quantum Chemical Calculations of Nitrosamine Activation and Deactivation Pathways for Carcinogenicity Risk Assessment

**Andreas H. Göller^1 *^, Sandra Johanssen^2^, Adam Zalewski^3^, Verena Ziegler^3^**

1 Computational Molecular Design, Bayer AG, Pharmaceuticals, Wuppertal, Germany

2 Industrial Chemicals & Marketed Products, Bayer AG, Pharmaceuticals, Berlin, Germany

3 Genetic and Computational Toxicology Bayer AG, Pharmaceuticals, Berlin, Germany

*** Correspondence:**

Andreas H. Göller

* andreas.goeller@bayer.com

**Bond distances and angles defining the transition states of the reaction pathways**

Table **T1** provides the bond distances and **T2** provides the bond angles defining the transition state structures of the 9 compounds. Figure F1 shows the definitions of the measures based on the example of the **NPIP** transition states. General observation is, that the larger and the more sterically constrained the N-nitrosamine, the longer the carbon-nitrogen distance for the diazonium ion-DNA adduct respectively the carbon-nitrogen distance for the carbenium ion-DNA adduct. This is also reflected in the angles N-C…N and C-C…N between the diazonium ion-carbon bond and the ring nitrogen of **5G** and the analogous C-C bond of the carbenium ion **4** and the ring nitrogen of **5G**. The other geometrical features are mostly conserved between the molecules’ transition states. The only other exception is the position of the transferred hydrogen in **1-2_TS**, that is on the reactant side for BBNA and on the product side for the other TS.

a)
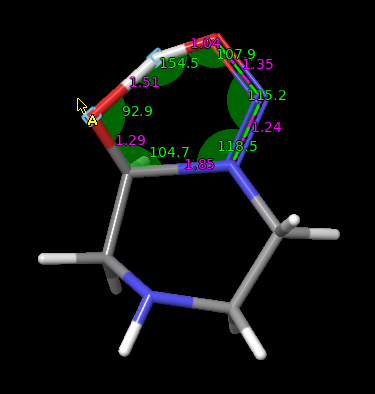
 b)
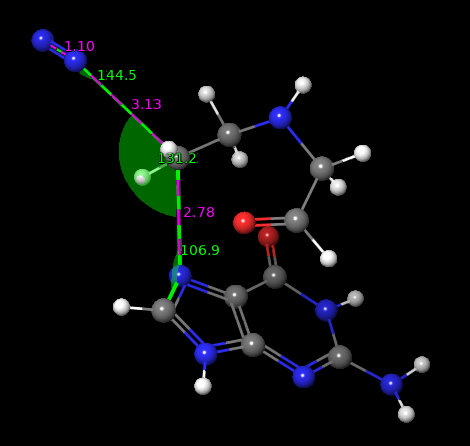


c)
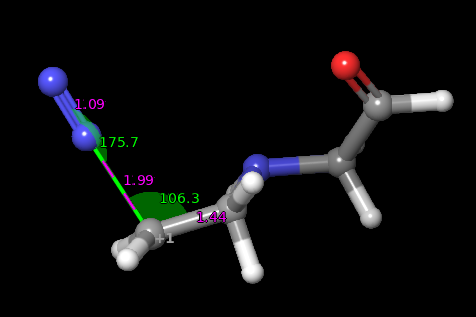
 d)
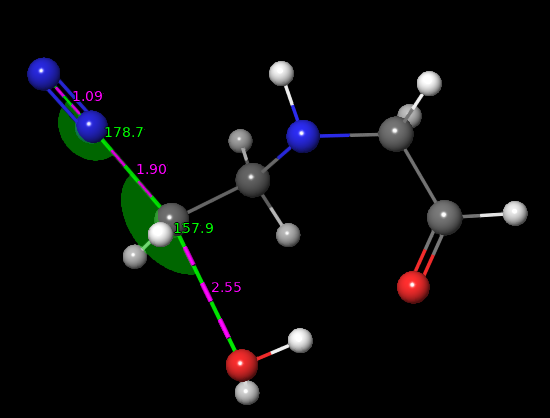


e)
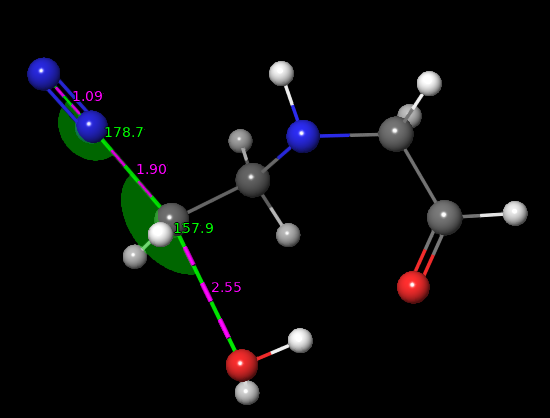


Figure F1: Definition of bond distances and angles for the five transition state structures of NPIP. Values are given in tables T1 and T2 for the 9 molecules.

|  |  | **NDMA** | **NPIP** | **NTBA** | **BBNA** | **NPZ** | **NMA** | **2-NMPY** | **3-NMPY** | **4-NMPY** |
| --- | --- | --- | --- | --- | --- | --- | --- | --- | --- | --- |
| **TS 1-2** | H-O | 1.47 | 1.44 | 1.58 | 1.28 | 1.51 | 1.56 | 1.53 | 1.54 | 1.56 |
|  | O-C | 1.28 | 1.3 | 1.28 | 1.32 | 1.29 | 1.28 | 1.28 | 1.28 | 1.28 |
|  | C-N | 1.81 | 1.85 | 1.88 | 1.9 | 1.85 | 1.85 | 1.85 | 1.84 | 1.85 |
|  | N=N | 1.26 | 1.24 | 1.23 | 1.25 | 1.24 | 1.25 | 1.25 | 1.25 | 1.25 |
|  | N=O | 1.33 | 1.34 | 1.36 | 1.33 | 1.35 | 1.34 | 1.34 | 1.34 | 1.34 |
|  | O…H | 1.06 | 1.06 | 1.02 | 1.14 | 1.04 | 1.03 | 1.03 | 1.03 | 1.03 |
|  |  |  |  |  |  |  |  |  |  |  |
| **TS 3-5G** | N~N | 1.1 | 1.09 |  | 1.09 | 1.1 | 1.1 | 1.09 | 1.09 | 1.09 |
|  | N-C | 1.71 | 1.83 |  | 1.77 | 3.13 | 2.37 | 2.09 | 2.29 | 1.84 |
|  | C…N | 2.41 | 2.59 |  | 3.94 | 2.78 | 2.88 | 3.68 | 3.43 | 2.23 |
|  |  |  |  |  |  |  |  |  |  |  |
| **TS 3-6** | N~N | 1.1 | 1.09 |  |  | 1.09 |  |  |  |  |
|  | N-C | 1.85 | 1.9 |  |  | 1.9 |  |  |  |  |
|  | C…O | 2.17 | 2.56 |  |  | 2.55 |  |  |  |  |
|  |  |  |  |  |  |  |  |  |  |  |
| **TS 3-4** | N~N |  | 1.09 |  | 1.09 | 1.09 |  |  |  |  |
|  | N...C |  | 2.06 |  | 1.78 | 1.99 |  |  |  |  |
|  | C-C |  | 1.44 |  | 1.49 | 1.44 |  |  |  |  |
|  |  |  |  |  |  |  |  |  |  |  |
| **TS 4-5G** | C…N |  | 3.1 |  |  | 2.27 |  |  |  |  |

Table T1: Bond lengths in Angstrom for the transition state structures from the reaction energy profiles of the nine compounds. Figure F1 shows the definition of the respective bonds.

|  |  | **NDMA** | **NPIP** | **NTBA** | **BBNA** | **NPZ** | **NMA** | **2-NMPY** | **3-NMPY** | **4-NMPY** |
| --- | --- | --- | --- | --- | --- | --- | --- | --- | --- | --- |
| **TS 1-2** | O-H-O | 154.8 | 156.4 | 155.2 | 158.5 | 154.5 | 155.5 | 155.7 | 155.4 | 155.5 |
|  | H-O-C | 97.1 | 95 | 94.1 | 100.4 | 92.9 | 94.5 | 95.6 | 94.9 | 94.5 |
|  | O-C-N | 107.4 | 104.3 | 107.5 | 101.2 | 104.7 | 108 | 107.4 | 107.7 | 108 |
|  | C-N=N | 120.7 | 118.6 | 120.4 | 118.9 | 118.5 | 120.9 | 121.2 | 121.1 | 120.9 |
|  | N=N=O | 115.3 | 115.3 | 115.5 | 116 | 115.2 | 115.1 | 115.1 | 115.1 | 115.1 |
|  | N-O…H | 108.6 | 107.7 | 109.1 | 106.7 | 107.9 | 109.5 | 109.4 | 109.5 | 109.5 |
|  |  |  |  |  |  |  |  |  |  |  |
| **TS 3-5G** | N~N-C | 173.8 | 179.2 |  | 178.8 | 144.5 | 153.2 | 170.1 | 169.5 | 169.4 |
|  | N-C…N | 175.5 | 158.6 |  | 135.5 | 131.2 | 75.6 | 80 | 80.6 | 79.3 |
|  | C…N=C8 | 142.1 | 121.9 |  | 99.7 | 106.9 | 98.3 | 97.6 | 84.9 | 120 |
|  |  |  |  |  |  |  |  |  |  |  |
| **TS 3-6** | N~N-C | 179.9 | 177.8 |  |  | 178.7 |  |  |  |  |
|  | N-C…O | 177.7 | 157.5 |  |  | 157.9 |  |  |  |  |
|  |  |  |  |  |  |  |  |  |  |  |
|  |  |  |  |  |  |  |  |  |  |  |
| **TS 3-4** | N~N...C |  | 178.1 |  | 177.8 | 175.7 |  |  |  |  |
|  | N...C-C |  | 106.6 |  | 103.2 | 106.3 |  |  |  |  |
|  |  |  |  |  |  |  |  |  |  |  |
|  |  |  |  |  |  |  |  |  |  |  |
| **TS 4-5G** | C-C…N |  | 95.8 |  |  | 105.1 |  |  |  |  |
|  | C…N=C8 |  | 102.2 |  |  | 120.7 |  |  |  |  |

Table T1: Bond angles for the transition state structures from the reaction energy profiles of the nine compounds. Figure F1 shows the definition of the respective angles.
